# The Role of Endothelin-1 and Endothelin Receptor Antagonists in Inflammatory Response and Sepsis

**DOI:** 10.1007/s00005-014-0310-1

**Published:** 2014-10-07

**Authors:** Agata Kowalczyk, Paulina Kleniewska, Michal Kolodziejczyk, Beata Skibska, Anna Goraca

**Affiliations:** 1 Chair of Experimental and Clinical Physiology, Department of Cardiovascular Physiology, Medical University of Lodz, Mazowiecka 6/8, 92-215 Lodz, Poland; 2 Chair of Applied Pharmacy, Faculty of Pharmacy, Medical University of Lodz, Lodz, Poland

**Keywords:** Endothelins, Sepsis, Inflammation, Reactive oxygen species, Endothelin receptor antagonists

## Abstract

Endothelin-1 (ET-1) is a potent endogenous vasoconstrictor, mainly secreted by endothelial cells. It acts through two types of receptors: ETA and ETB. Apart from a vasoconstrictive action, ET-1 causes fibrosis of the vascular cells and stimulates production of reactive oxygen species. It is claimed that ET-1 induces proinflammatory mechanisms, increasing superoxide anion production and cytokine secretion. A recent study has shown that ET-1 is involved in the activation of transcription factors such as NF-κB and expression of proinflammatory cytokines including TNF-α, IL-1, and IL-6. It has been also indicated that during endotoxaemia, the plasma level of ET-1 is increased in various animal species. Some authors indicate a clear correlation between endothelin plasma level and morbidity/mortality rate in septic patients. These pathological effects of ET-1 may be abrogated at least partly by endothelin receptor blockade. ET-1 receptor antagonists may be useful for prevention of various vascular diseases. This review summarises the current knowledge regarding endothelin receptor antagonists and the role of ET-1 in sepsis and inflammation.

## Pathogenesis of Sepsis

Sepsis is defined as a systemic inflammatory response syndrome, most commonly provoked by severe bacterial infection (Naito et al. 2014; Sagy et al. 2013; Zhang et al. 2014) This critical condition, with a mortality rate of about 50–80 %, is characterised by hyperthermia or hypothermia, tachypnea, tachycardia, leucocytosis or leucopenia, with immature neutrophils, and organ dysfunction due to impaired tissue perfusion (Sagy et al. 2013). Endotoxic shock is also associated with pulmonary hypertension, systemic hypotension and cardiac dysfunction (Forni et al. 2005). The mechanisms underlying the pathogenic effects of sepsis are still not completely understood.

The primary cause of escalated inflammatory response in septic shock is the presence of bacterial toxins. These include the lipopolysaccharide (LPS) endotoxin, which is a compound of a Gram-negative bacterial cell wall and an exotoxin (superantigen) from Gram-positive bacteria. When released into the blood, these bacterial products induce macrophages to secrete large amounts of inflammatory cytokines like tumour necrosis factor (TNF)-α, interleukin (IL)-1, IL-6, and IL-8, by the activation of signalling cascades such as nuclear factor (NF)-κB and mitogen-activated protein kinase (MAPKs) pathways. Exotoxins also activate T-lymphocytes to produce proinflammatory mediators, IL-2 and interferon γ, which stimulate inducible nitric oxide synthase (iNOS) to produce nitric oxide (NO). Together with IL-2, they also intensify the release of IL-1 and TNF-α from macrophages (Roth and De Souza 2001; Sagy et al. 2013; Zhang et al. 2014). Furthermore, septic shock is also associated with an increased level of platelet-activating factor, thromboxane A2, leukotrienes, macrophage inflammatory protein-1, prostaglandin E2 (PGE2), cyclooxygenase (COX)-2 mRNA and endothelin-1 (ET-1) (Dilshara et al. 2014; Jesmin et al. 2014; Keller et al. 2006; Roth and De Souza 2001). Under pathological conditions, endotoxin stimulates endothelin systems to release large amounts of endothelin into the blood stream. Likewise, the levels of ETA and ETB receptor mRNA are also elevated in some tissues (Forni et al. 2005). LPS-induced sepsis impairs the integrity of the endothelial barrier resulting in endothelial cell injury, which contributes to greater permeability of endothelial cells and impaired homeostasis, and induces the release of cytokines and reactive oxygen species (ROS). Plasma levels of ET-1 are elevated in septic patients and are associated with the severity of the illness. Some authors indicate a clear correlation between endothelin plasma level and morbidity and mortality in septic patients (Pan et al. 2012).

## The Endothelin System

The endothelins are a family of 21 amino acid peptides with three distinct isoforms: ET-1, ET-2 and ET-3. ET-1 is the most abundant and the best described isoform. ET-2 and ET-3 were identified later and are not as well-studied (Motte et al. 2006; Yanagisawa et al. 1988). ET-1 is produced in a variety of cells (Table 1) and there are many factors which regulate its secretion (Table 2). Physical and chemical stimulants activate ET-1 gene expression in endothelial cells by the DNA binding of transcription factors such as activator protein-1, GATA-2, smad, hypoxia inducible factor-1 and NF-κB (Rodriguez-Pascual et al. 2003; Wanecek et al. 2000; Wort et al. 2009).

**Table 1:** Expression of endothelin receptors and cells producing ET-1

	Cardiovascular system	Urinary system	Nervous system	Immune system and skin	Respiratory system	Other tissues and cells
Cells producing ET-1	Endothelium, VSMCs, cardiomiocytes^c, g^	Renal medulla^b^	Neurons^c^	Macrophages, leucocytes^g^, mast cells^a^, Kupffer cells^e^	Tracheal epithelium^b^, airway epithelial cells^c^	Fibroblasts^g^, hepatic sinusoids^e^
Cells expressing receptor ETA	VSMCs, cardiomiocytes^c^, nuclear membranes in human aortic VSMCs	Glomerular capillaries, medullary collecting ducts^c^	Neurons^c^, vagus nerve^h^	Melanocyte, keratinocytes^c^		Adipocytes, osteoblasts, hepatocytes, liver stellate cells, reproductive system cells^c^
Cells expressing receptor ETB	Endothelium, VSMCs^f^, coronary vasculature, aorta, atrioventricular conducting tissue, atrial and ventricular myocardium^c^, nuclear membranes in human aortic VSMCs^d^	Renal tubules, glomerular capillaries, medullary collecting ducts^c^	Brainstem neurons and glia, sympathetic nervous system^c^, vagus nerve^h^			Various endocrine tissues, osteoblasts, hepatocytes^c^

^a^ Ehrenreich et al. (1992), ^b^ Endo et al. (1992), ^c^ Hynynen and Khalil (2006), ^d^ Lima et al. (2011), ^e^ Liu et al. (1997), ^f^ Motte et al. (2006), ^g^ Ohkita et al. (2012), ^h^ Rodriguez et al. (2013)

**Table 2:** Factors, which stimulate and inhibit release of ET-1

Factors stimulating release of ET-1	Factors inhibiting release of ET-1
Low shear stress^j^	High shear stress^f^
Adrenalin^i^	Nitric oxide^e^
Thrombin^e^	Prostacyclin^c^
Angiotensin II^5^	Heparin^e^
Hypoxia^e^	Prostaglandin^e^
Vasopressin^c^	Atrial natriuretic peptide^c^
Endotoxin (LPS)^g^	
IL-1^d^	
Transforming growth factor-β^c^	
TNF-α^c^	
Insulin^e^	
Free radicals^e^	
Cardiotrophin-1^e^	
Homocysteine^a^	
IL-6^h^	
Calcium ions^b^	

^a^ Duan et al. (2008), ^b^ Hukovic and Hadziselimovic (1998), ^c^ Hynynen and Khalil (2006), ^d^ Maemura et al. (1992), ^e^ Motte et al. (2006), ^f^ Shao et al. (2011), ^g^ Sugiura et al. (1989), ^h^ Yamashita et al. (1993), ^i^ Yanagisawa et al. (1988), ^j^ Yoshizumi et al. (1989)

**Fig. 1:**
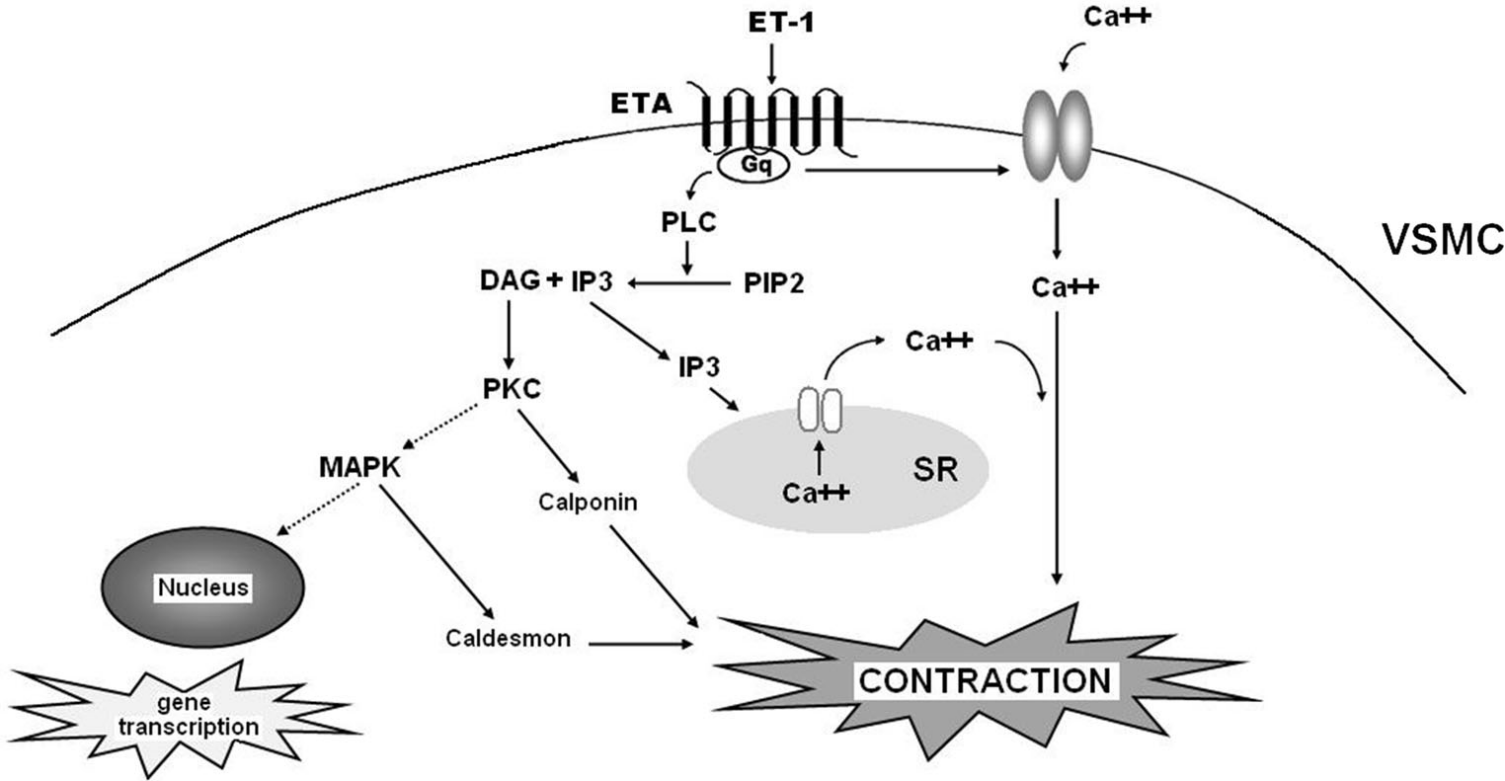
ETA receptor-mediated VSMC signalling pathways. Activation of the ETA receptor stimulates phospholipase C (PLC) to generate inositol 1,4,5-trisphosphate (IP_3_) and diacylglycerol (DAG) from phosphatidylinositol 4.5-bisphosphate (PIP_2_). IP_3_ induces Ca^2+^ outflow from intracellular stores in sarcoplasmic reticulum (SR). Furthermore, the ETA receptor acts on nonselective plasmalemmal Ca^2+^ channels causing Ca^2+^ input from the extracellular space. Consequently, increased concentrations of Ca^2+^ leads to the contraction of VSMC. The activated ETA receptor also stimulates cell growth. Production of DAG activates protein kinase C (PKC), which is responsible for the mitogenic function of endothelin, and which also induces a Ca^2+^-independent pathway of VSMC contraction involving calponin phosphorylation. PKC affects gene transcription through activation of the Ras/Raf/MEK/MAPK cascade. MAPK phosphorylates caldesmon, which increases VSMC contraction (Hynynen and Khalil 2006; Khalil 2011; Lima et al. 2011)

Two types of endothelin receptors are found in mammals, ETA and ETB receptors, which belong to the G protein-coupled receptors family. ETA receptors are located mostly in vascular smooth muscle cells (VSMC), where they are responsible for potent vascular contraction (Fig. 1), cell proliferation and a proinflammatory effect. ETB receptors include two subtypes: ETB_1_, which is expressed on endothelial cells and evokes NO-mediated vasodilation, and ETB_2_, also present in VSMC, which causes contraction (Hynynen and Khalil 2006; Yanagisawa et al. 1988). Stimulation of ETB_1_ receptors also results in the release of other vasodilatory factors such as prostacyclin (PGI_2_) and endothelium-derived hyperpolarizing factor. Furthermore, it is suggested that endothelial ETB receptors take part in ET-1 clearance, but findings are not unequivocal (Hynynen and Khalil 2006; Kawanabe and Nauli 2011; Ohkita et al. 2012).

So far, ETA and ETB receptors have been detected in many cell types other than blood vessels (Table 1), but predominantly in cardiovascular tissues (Hynynen and Khalil 2006). Endothelins, through their receptors, exert an influence on the function of many organs like the heart, the kidneys, the lungs and the liver (Lima et al. 2011; Rodriguez et al. 2013). Apart from participating in the regulation of vascular tone, endothelins take part in vascular, myocardial and bone remodelling, inhibition of apoptosis and salt-water retention. Furthermore, endothelins can contribute to bronchoconstriction, angiogenesis and neuropathic pain (Rodriguez et al. 2013; Thakkar et al. 2006).

## ET-1 and ROS

It is known that ET-1 stimulates the production of ROS, primarily superoxide anions (O_2_^−^), and consequently leads to the development of oxidative stress. When given intravenously, the peptide initially causes vasodilation of blood vessels followed by their subsequent long lasting contraction, resulting in ischemia of internal organs and the dysfunction of the endothelium. These changes can lead to abundant ROS generation (Dong et al. 2005; Loomis et al. 2005; López-Sepúlveda et al. 2011; Thakali et al. 2005). Some studies have shown the oxidative stress caused by ET-1 to be associated with augmentation of lipid peroxidation and reduction of intracellular GSH (glutathione) and SH groups (Scalera et al. 2002; Viswanatha Swamy et al. 2011). According to the literature, both the ETA (Elmarakby et al. 2005) and ETB receptors (Dong et al. 2005) mediate ROS generation. Callera et al. (2003) demonstrated that elevated concentrations of ET-1 induce the synthesis of O_2_^−^ through ETA receptors. Xu et al. (2003) report a significant reduction in lipid peroxidation products content in the ischemic myocardium after applying BQ123, a blocker of the ETA receptor. Other authors note a reduction of lipid peroxidation in various organs during oxidative stress when using this blocker (Briyal et al. 2011). BQ123 has been found to block increased production of O_2_^−^ in ET-1-induced oxidative stress in the arteries and veins of patients with coronary artery disease (Cerrato et al. 2012), has been associated with a significant increase in the concentration of total glutathione and superoxide dismutase (SOD) activity after application (Briyal et al. 2011), as well as a significant increase in the activity of the antioxidant enzymes SOD and catalase in cases of ET-1-induced oxidative stress (Ozdemir et al. 2006).

Studies on blocking ETB receptors are ambiguous. Wedgwood et al. (2001) note that the ETB receptor antagonist (Res-701-3) increased the production of H_2_O_2_ in the smooth muscle cell culture from the pulmonary artery, but not in endothelial cells. However, other studies indicate that the ETB receptor blockade reduces the ROS production in various tissues (Dai et al. 2004). Moreover, Piechota-Polanczyk et al. (2012) report a significant increase in the concentration of GSH, but insignificant increase in the ratio of GSH/GSSG in the rat heart after application of BQ788. On the other hand, Leonard et al. (2011, 2012) did not observe any changes in the concentration of glutathione in rats after administration of BQ788.

## The Proinflammatory Effect of ET-1

Several studies have demonstrated that ET-1 contributes to the development of inflammatory processes in the vascular wall. ET-1 has been found to be associated with an inflammatory response involving activation of transcription factors such as NF-κB and expression of proinflammatory cytokines including TNF-α, IL-1 and IL-6 (Yeager et al. 2012). These transcription factors and proinflammatory cytokines in turn stimulate ET-1 production (Virdis and Schiffrin 2003). Bellisai et al. (2011) report that ET-1 increases the synthesis of TNF-α in macrophages and monocytes. This cytokine enhances the inflammatory response by stimulating the chemotaxis and phagocytosis of macrophages, monocytes and neutrophils. Increased production of ROS in different types of cells occurs via the NF-κB, COX and NADPH oxidase-dependent pathways (Donate et al. 2012; Kleniewska et al. 2013; Piechota and Goraca 2011).

Recent studies have shown that the ETA receptor antagonist BQ123 has a beneficial influence on the concentration of TNF-α (Ozdemir et al. 2006). Ford et al. (2008) note that the ETA receptor blockade lowered the concentration of TNF-α in patients after bypass grafting via the antagonist BQ123. Chen et al. (2010) confirmed that BQ123 inhibited the expression of TNF-α and IL-1β in the lungs of rats during oxidative stress induced by intraperitoneal administration of an extract from the cigarette smoke. Moreover, an ETB receptor blockade may also have an influence on TNF-α synthesis. In a recent study by Tonari et al. (2012), BQ788 showed significant inhibition of the expression of TNF-α when applied to patients with damage to the optic nerve. However, Piechota-Polanczyk et al. (2012) report no significant reduction in the concentrations of TNF-α in the rat heart following the application of BQ788.

Furthermore, an excess amount of proinflammatory cytokines activates prostaglandin production during inflammatory responses in a number of cell types such as vascular endothelial and smooth muscle cells. Prostaglandins are synthetized by COX, which is also known as prostaglandin endopeptidase synthase. In this process, phospholipase A2 catalyses the release of arachidonic acid (AA) from membrane phospholipids, while COX catalyzes the conversion of AA into prostaglandins. There are two COX isoforms: COX-1 is constitutively expressed under normal conditions in the most tissues. This isoform takes part in regulating normal physiological responses and controls vascular homeostasis. COX-2 is not detectable in most normal cells and tissues, but its expression increases in inflammatory cells. Thus, COX-2 may play a crucial role in the development of various inflammatory responses including vascular inflammation. Recent studies have indicated that ET-1 induces COX-2 expression and PGE2 release by MAPKs and NF-κB (Lin et al. 2013).

ET-1 enhances the expression of adhesion molecules on vascular endothelial cells and stimulates the aggregation of polymorphonuclear neutrophils (PMNs) contributing to inflammation and endothelial dysfunction. Li et al. (2003) postulate that ET-1 stimulates the arterial vascular adhesion molecule-1 (VCAM-1) in hypertensive patients. VCAM-1 and the intracellular adhesion molecule-1 induce firm adhesion of inflammatory cells at the vascular surface (Blankenberg et al. 2003). PMNs may contribute to myocardial damage by releasing ROS, proteases and arachidonic acid metabolites (Hansen 1995). Oktar et al. (2000) indicate that ET-1 causes an accumulation of PMNs, oxidative stress, and mucosal dysfunction in the rat small intestine. Gonon et al. (2001) showed that the ET receptor blockade attenuates the accumulation of neutrophils and myeloperoxidase activity in the ischemic myocardium. It has been shown that the vascular injury caused by carotid artery ligation results in vascular inflammation and neointima formation. This action is attenuated in vascular endothelial ET-1-knockout mice (Anggrahini et al. 2009).

Increased ROS release contributes to endothelium dysfunction. Endothelial dysfunction occurs in cardiovascular diseases such as atherosclerosis. Increased expression of ET-1 was observed both in experimental models of atherosclerosis as well as in human atherosclerosis (Barton et al. 2003) and its level correlated with the severity of the atherosclerotic lesion. Haug et al. (1996) identify a higher expression of ET-1 in human VSMC harvested from human atherosclerotic coronary arteries than in cells from non-atherosclerotic arteries. ET-1 was associated with regions of the atherosclerotic plaque, particularly in regions with high macrophage content (Dashwood and Tsui 2011). It was shown that overexpression of ET-1 significantly increased the atherosclerotic lesion size in apolipoprotein E gene deleted mice (ApoE^−/−^) fed a high-fat diet. In this case, increased endothelial ET-1 expression enhances an increase in expression of genes associated with lipid synthesis in the vasculature and accelerates the progression of atherosclerosis (Simeone et al. 2011).

Li et al. (2013) have reported that ET-1 plays a role in the development of atherosclerosis and abdominal aortic aneurysm by decreasing high-density lipoprotein, increasing oxidative stress and monocyte/macrophage infiltration in both the aorta and aneurysms. So, plaque formation and endothelial function can be restored in the atherosclerosis model by the administration of ETA or dual ETA/ETB receptor antagonists. Moreover, it has been noted that tissue ET-1 concentration is more important than serum ET-1 in predicting atherosclerosis in patients with chronic kidney disease (Noshad et al. 2009). Recently, a study indicated that ethanolic extract of propolis inhibits atherosclerotic lesion formation in ApoE^−/−^ mice fed a high-fat diet, probably by regulating the inflammatory reaction and inhibiting ET-1 (Fang et al. 2013). Overexpression of ET-1, particularly in the endothelium of mice with atherosclerosis, is accompanied by a decrease in endothelial signalling pathways responsible for endothelium-dependent relaxation and an increase in the activity of sensitive voltage-dependent potassium channels (Mian et al. 2013). ROS are important physiological messengers in vascular cells and their overproduction contributes to the progression of atherosclerosis (Freund-Michel et al. 2013). ET-1 receptor antagonists may be useful for prevention of various vascular diseases (Kitada et al. 2009, 2012).

## ET-1 and Endothelin Receptor Antagonists in Sepsis

Recently, studies have addressed the role of endothelins and blockers of their receptors in the development of sepsis. During endotoxaemia, plasma endothelin level is increased in various species (Kaszaki et al. 1997; Pan et al. 2012; Piechota-Polańczyk and Gorąca 2012). In experimental models, endotoxin induces the expression of preproendothelin-1 mRNA in the lung and heart (Kaddoura et al. 1996). Infusion of ET-1 in septic shock contributed to the dysfunction of several vital organs such as liver, lung, heart and kidney (Fenhammar et al. 2011; Piechota-Polanczyk et al. 2012). It has been indicated that the infusion of ET-1 in humans causes cardiovascular changes in part resembling those observed during sepsis i.e. decreased cardiac output, vasoconstriction in the pulmonary artery, impairment of renal and splanchnic circulation (Bomberg et al. 2013; Ross 2012; Schuuring et al. 2013). It has been demonstrated in animal experiments that dual ETA/ETB endothelin blockade during endotoxaemia improves cardiopulmonary function, reduces pulmonary hypertension and lung injury and attenuates intestinal and liver microcirculatory dysfunction (Kuklin et al. 2005; Sánchez-Etayo et al. 2012). Also ETA receptor blockade alone improves the function of the lungs (Mercier et al. 2010), kidney (Rullman et al. 2010), heart (Vanêcková et al. 2005) and aorta (Tirapelli et al. 2008).

Endothelin receptor antagonists (ERAs) are a new, promising class of medicines which block the ETA and ETB endothelin receptors with varying degrees of selectivity. They form a large group consisting of nearly 40 or more compounds, and part of them is currently under investigation (Hynynen and Khalil 2006; Khalil 2011; Motte et al. 2006) as potential therapeutic objects in clinical trials (see Table 3). ERAs act on various pathophysiological mechanisms (Table 4), three of which recommended for treatment of pulmonary arterial hypertension, bosentan, ambrisentan and macitentan, are already on the world pharmaceutical market (Motte et al. 2006; Patel and McKeage 2014). However, the nonselective ERAs, bosentan and tezosentan, and selective antagonists, BQ123 and BQ788, are currently receiving the most attention with regard to the effect of ERAs on the progression of sepsis.

**Table 3:** Main ERAs in clinical and preclinical trials

ERA	Selectivity	Negative results in	Positive results in
Ambrisentan (Letairis^®^, USA; Volibris^®^, EU)	ETA	Idiopathic pulmonary fibrosis (phase I clinical study)^22^	Treatment of pulmonary arterial hypertension associated with spironolactone (ARIES trials)^17^, therapy of pulmonary arterial hypertension in children (phase 0 clinical study)^30^
Atrasentan (ABT-627, A-147627)	ETA	Metastatic hormone-refractory prostate cancer (phase III clinical study)^2^	Diabetic nephropathy: reduced albuminuria (phase II clinical study)^1, 13^
		Castration-resistant prostate cancer and bone metastases (phase III clinical study)^21^	Cerebrovascular dysfunction in diabetes: improved cerebrovascular relaxation (preclinical study)^15^
			Early atherosclerosis: improved endothelial function and inhibited plaque progression (phase I clinical study)^24, 33^
Avosentan (SPP 301)	ETA	Diabetic and non-diabetic chronic kidney disease (ASCEND trial)^12^	Glaucoma (preclinical study)^14, 31^
Bosentan (Tracleer^®^)	ETA/ETB	Pulmonary hypertension associated with fibrotic idiopathic interstitial pneumonia (phase 0 clinical study)^5^	Ovarian ischaemia/reperfusion (I/R) injury: limited oxidative damage and I/R injury (preclinical study)^27^
			Rheumatoid arthritis: antinociceptive and anti-inflammatory activity (preclinical study)^9^
			Diabetes: improved learning and memory abilities (preclinical study)^29^
Clazosentan (RO 61-7790)	ETA	Prevention of occurrence of cerebral vasospasm after aneurysmal subarachnoid haemorrhage: controversial results (CONSCIOUS-2 and halted CONSCIOUS-3 trials)^16, 28^	
Darusentan (LU-135252)	ETA	Resistant hypertension: significantly decreased blood pressure, but serious adverse events (DORADO trial)^7^	
Macitentan (Opsumit^®^)	ETA/ETB	Idiopathic pulmonary fibrosis (MUSIC trial)^23^	Ovarian cancer: inhibited progression, reduced tumour weight (preclinical study)^10, 11^
Tezosentan (RO 61-0612)	ETA/ETB	Right ventricular failure (TACTICS trial)^6, 19^	Ischemic cardiomyopathy: protective properties (preclinical study)^25^
		Type 2 hepatorenal syndrome (phase 0 clinical study)^32^	
Zibotentan (ZD4054)	ETA	Metastatic and non-metastatic hormone- and castration-resistant prostate cancer (phase III clinical study)^18, 20, 26^	Colorectal cancer (preclinical study)^8^
		Ovarian cancer (phase II clinical study)^4^	
		Non-small cell lung cancer (phase II clinical study)^3^	

^1^ Braun et al. (2012), ^2^ Carducci et al. (2007), ^3^ Chouaid et al. (2011), ^4^ Cognetti et al. (2013), ^5^ Corte et al. (2014), ^6^ Denault et al. (2013), ^7^ Grassi (2011), ^8^ Haque et al. (2013), ^9^ Imhof et al. (2011), ^10^ Kim et al. (2011), ^11^ Kim et al. (2012), ^12^ Kohan and Pollock (2013), ^13^ Kohan et al. (2011), ^14^ Konieczka et al. (2011), ^15^ Li et al. (2011), ^16^ Macdonald et al. (2013), ^17^ Maron et al. (2013), ^18^ Miller et al. (2013), ^19^ Motte et al. (2006), ^20^ Nelson et al. (2012), ^21^ Quinn et al. (2013), ^22^ Raghu et al. (2013a), ^23^ Raghu et al. (2013b), ^24^ Reriani et al. (2010), ^25^ Ryu et al. (2009), ^26^ Schelman et al. (2011), ^27^ Sengul et al. 2013, ^28^ Shen et al. (2013), ^29^ Singh et al. (2014), ^30^ Takatsuki et al. (2013), ^31^ Wang et al. (2011), ^32^ Wong et al. (2008), ^33^ Yoon et al. (2013)

**Table 4:** The effects of endothelin receptor blockers on various pathophysiological mechanisms in sepsis—summary

Blocker	Selectivity	Effect	References
BQ123	ETA	Reduction in lipid peroxidation products, TNF-α and H_2_O_2_ concentration	Briyal et al. (2011), Piechota et al. (2011), Piechota-Polańczyk and Gorąca (2012), Xu et al. (2003)
		Increase in the concentration of total glutathione, elevated SOD and catalase activity	Ozdemir et al. (2006)
		Decrease in the concentration of TNF-α and inhibition of TNF-α expression	Chen et al. (2010), Ford et al. (2008), Ozdemir et al. (2006)
		Inhibition of IL-1β expression	Chen et al. (2010)
Bosentan	ETA/ETB	Decrease in organ injury, improved microcirculatory blood flow in splanchnic organs and in peripheral tissues	Iskit et al. (2004), Krejci et al. (2003)
		Inhibition of the up-regulation of ET-1, iNOS, and COX-2 mRNA	Keller et al. (2006)
Tezosentan	ETA/ETB	Improved cardiopulmonary function, reduced pulmonary hypertension, reduced lung, liver, kidney and spleen injury and attenuated intestinal, renal and liver microcirculatory dysfunction	Chin et al. (2002), Kuklin et al. (2005)
BQ788	ETB	Reduction in the ROS production in various tissues	Dai et al. (2004)
		Increase of mean arterial pressure	Nitescu et al. (2008)
		Protective and anti-inflammatory effects in the brain tissue	Naito et al. (2014)

## Bosentan

In 2001, bosentan (Tracleer^®^) became the first ERA to be registered in the USA as an oral medicine for patients with pulmonary arterial hypertension of functional class III/IV (Dupuis and Hoeper 2008; Motte et al. 2006). Many studies have shown a significant improvement in functional class and exercise capacity, as well as haemodynamic, Doppler and echocardiographic parameters after treatment with bosentan. Despite having side effects such as increases in liver transaminase content, headache, peripheral oedema, dizziness, nasal congestion and nausea, bosentan therapy is generally regarded as beneficial (Montani et al. 2013; Motte et al. 2006). Bosentan also has been found to exert a positive therapeutic influence on the treatment of systemic sclerosis (scleroderma) and other disorders (Cozzi et al. 2013) (Table 3).

A common feature of sepsis is microcirculatory dysfunction. However, Krejci et al. (2003) note improved microcirculatory blood flow in the pancreas, gastric, skeletal muscle and colon mucosa of septic pigs treated with bosentan. Further, Iskit et al. (2004) report that bosentan at a dose of 30 mg/kg b.w. decreases caecal ligation and reduce liver, kidney and spleen injury, improving survival (*p* < 0.05) in a mouse model of polymicrobial sepsis. They also suggest that antagonism of endothelin receptors during the hypodynamic phase of septic shock gives much better results. More detailed data provided by Keller et al. (2006) confirm that the administration of LPS results in increased expressions of ET-1, iNOS, and COX-2 mRNA. They also report a significant inhibition of the up-regulation of ET-1, iNOS, and COX-2 mRNA after treatment of rats with 30 mg/kg b.w. bosentan, thus demonstrating its anti-inflammatory and therapeutic properties.

## Tezosentan

The first study concerning the effects of tezosentan on the cardiovascular system in sepsis was conducted by Chin et al. (2002). During endotoxaemia, endothelin antagonism with this ERA maintained renal and cardiac functions, preventing decreases in cardiac index, renal blood flow, glomerular filtration rate and increased systemic vascular resistance in neonatal piglets (Chin et al. 2002). Further research demonstrated that tezosentan (10 mg/kg b.w) prevents mesenteric ischemia in septic mice. It significantly (*p* = 0.0046) attenuated decreases in mesenteric blood flow and limited injury to organs such as the liver, kidney and spleen. However, in this study, despite what was expected, tezosentan did not reduce ROS generation (Erdem et al. 2007). Other sepsis studies on pigs have brought similar effects, confirming that application of tezosentan results in improved intestinal and renal microcirculation, contributes to increased portal vein flow and decreased pulmonary capillary wedge pressure, and preserves cardiac index. Moreover, pH and arterial lactate values were better compared to the control (Andersson et al. 2008; Fenhammar et al. 2008). It is also significant that this dual blocker had no influence on TNF-α, IL-6 or IL-10 plasma levels, nor angiotensin II or aldosterone plasma concentration, in this model of sepsis (Fenhammar et al. 2008). A recent investigation using tezosentan and the selective ETA antagonist, TBC3711 revealed no improvement in the liver and ileum microvascular blood flow during selective antagonism, but it showed marked amelioration after tezosentan. These findings highlight the special role of the ETB receptor in mediating the microcirculatory failure in this area (Andersson et al. 2010).

## BQ123 and BQ788

Selective endothelin receptor blockers like BQ123 and BQ788 have also been used in sepsis studies: the former being an ETA receptor antagonist and the latter an ETB receptor blocker. Hirata and Ishimaru (2002) report that BQ123 does not increase survival in a rat septic shock model, due to lack of improvement in LPS-induced profound hypotension. However, a dual blockade of endothelin receptors helped maintain normal mean arterial pressure. The authors also suggest that this effect does not depend on iNOS-derived NO (Hirata and Ishimaru, 2002). Similarly, Nitescu et al. (2008) note that while BQ123 did not prevent endotoxin-induced hypotension, BQ788 did, by increasing mean arterial pressure in septic rats. Moreover, marked decrease (*p* < 0.05) of renal blood flow was observed in the group treated with both BQ788 and LPS, which may indicate that the ETB receptor is responsible for renal vasodilation and maintenance of normal renal blood flow (Nitescu et al. 2008).

Some authors suggest that oxidative stress in sepsis is mediated by ET-1 and ETA receptors. Studies on a rat model have shown that LPS markedly elevates lipid peroxidation products, TNF-α and H_2_O_2_ concentration in the lung, and BQ123 administration resulted in a distinct decrease of these parameters (*p* < 0.05), except for lipid peroxidation products level, which remained elevated. This ETA blocker also reduced TNF-α level in lung and plasma. Furthermore, a lower dose of BQ123 (0.5 mg/kg) was found to be more effective than higher dose of 1 mg/kg, and also prevented lung oedema development (*p* < 0.01) (Piechota et al. 2011; Piechota-Polańczyk and Gorąca 2012). On the other hand, the same study based on heart tissue demonstrated a significant reduction in lipid peroxidation products and TNF-α, but not in H_2_O_2_ concentration, after BQ123 administration in experimental sepsis (Piechota-Polanczyk et al. 2012). These performance differences are probably associated with different antioxidant enzymes activities in the lung and heart.

Moreover, ETB receptor antagonism with BQ788 also reduced amplified amount of ROS, lowering lipid peroxidation products and H_2_O_2_ concentration. However, BQ788 did not influence increased concentration of TNF-α and neither BQ123 nor BQ788 affected LPS-induced activation of NF-κB pathway. However, Naito et al. (2014) note the protective effects of BQ788, including inhibition of neuroblast apoptosis, c-FOS expression, number of reactive microglia and distinctly diminished TNF-α level in mouse brain tissue during sepsis. These findings may suggest that in the heart, the ETA receptor is more involved in secretion of TNF-α than ETB. Furthermore, presumably both endothelin receptors in heart tissue stimulate ROS generation by some other signalling pathways than NF-κB. Nevertheless, the blockage of ETA receptor reduces ROS production and improves tissue antioxidant properties (Piechota-Polanczyk et al. 2012). These findings also confirm to some extent that ET-1 is strongly involved in the pathogenesis of septic shock, but the close links between factors inducing sepsis and increased production of ET-1 are still unclear.

## Towards the End

A very recent study has revealed that the oedema-promoting effects of ET-1 might be related to augmented level of heparin-binding protein (HBP) accompanying sepsis. HBP, also termed CAP37 or azurocidin is a protein released from neutrophils, which induces vascular hyperpermeability and contributes to oedema formation during endotoxaemia. Until now, it was believed that ET-1 induces vascular leakage, but the mechanisms of this action were not established. Using a porcine sepsis model, Persson et al. (2014) report a significant decrease of HBP plasma level, as well as reduced pulmonary oedema, in animals treated with tezosentan compared to those who received ET-1 or the ETB receptor agonist sarafotoxin 6c, both of which caused a dose-dependent increase in HBP levels similar to those observed in sepsis. These findings suggest that stimulation of both endothelin receptors activates reactions which lead to augmented secretion of HBP and this process may be abolished by nonselective ERA (Persson et al. 2014).

More than 20 years ago, ET-1 was identified as the first endothelium-derived contracting factor. Shortly after, two receptor subtypes, ETA and ETB, were discovered, which permitted the development of orally active ET-1 receptor antagonists. One of the first was the nonselective ETA/ETB-receptor antagonist, bosentan (Tracleer^®^), available to patients in 2001. Since then, although many ERAs have been described, not all of them are in clinical use. A novel ERA, macitentan (Opsumit^®^), is a very potent nonslective blocker, with high lipophilicity (Montani et al. 2013) “sustained receptor binding and enhanced tissue penetration properties” (Sidharta et al. 2013). It is the newest and safest registered ERA (Patel and McKeage 2014), but its specific properties have not yet been studied in condition of sepsis. Similarly, a potent ETA receptor antagonist, ambrisentan (Letairis^®^, Volibris^®^) may present favourable effects in sepsis (Montani et al. 2013; Elshaboury and Anderson 2013).

New applications for this group of medicines still remain an interesting subject of study. Antibiotics, fluids and vasopressors are most commonly used in the treatment of sepsis. Recent anti-inflammatory strategies such as high-dose corticosteroids, anti-TNF-*α*, IL-1-based therapies or activated protein C, have proved to be disappointing (Xie et al. 2014). Effective clinical applications of endothelin receptor antagonists need a more thorough understanding of the physiology and pathophysiology of the ET-1 system. Endothelins, HBP and other, yet unidentified, factors involved in the bacterial toxin activation of the endothelin system might represent the main target for sepsis therapy in the future.
